# Pharmacokinetics and safety of single and repeat doses of ceftibuten in healthy participants: a phase 1 dose escalation study

**DOI:** 10.1128/aac.00087-25

**Published:** 2025-08-05

**Authors:** Mary Beth Dorr, Carlos Fernando de Oliveira, Jeroen van de Wetering, Kathryn Lowe, Philip Sabato, Gregory Winchell, Hongzi Chen, Paul C. McGovern

**Affiliations:** 1 VenatoRx Pharmaceuticals Inc, Malvern, Pennsylvania, USA; 2 PRA Health Sciences 107593, Groningen, the Netherlands; 3 Winchell Pharma Consulting, LLC, Norristown, Pennsylvania, USA; University of California San Francisco, San Francisco, California, USA

**Keywords:** ceftibuten, pharmacokinetics, clinical trial, phase 1, drug safety

## Abstract

Ledaborbactam etzadroxil, the prodrug of the active β-lactamase inhibitor ledaborbactam, is being developed in combination with ceftibuten to treat serious infections caused by drug-resistant Enterobacterales. This study evaluated the safety and pharmacokinetics of ceftibuten in healthy adults at anticipated higher doses required, in combination with ledaborbactam etzadroxil, to treat Enterobacterales infections. Thirty-six participants (*n* = 12 per cohort [ceftibuten *n* = 9, placebo *n* = 3]) received a single oral dose of ceftibuten (400, 800, or 1,200 mg) or matched placebo on day 1. Following a one-day washout, the same participants received repeat oral doses of ceftibuten (400 mg once daily, 400 mg every 12 hours [q12h], or 400 mg q8h) for 10 days. Of the 36 participants, 25 (ceftibuten 67%, placebo 78%) reported 65 treatment-emergent adverse events (TEAEs). Nausea (22%), headache (15%), and fatigue (15%) were the most reported TEAEs among ceftibuten-treated participants. No participant experienced serious adverse events or discontinuations due to TEAEs. In both single- and multiple-dose cohorts, cis-ceftibuten isomer exposure was dose-proportional for areas under the curve (AUCs) but less than dose-proportional for maximum concentrations (*C*_max_). Low levels of cis-ceftibuten accumulation were observed at steady state, with accumulation ratios of 1.06, 1.09, and 1.24 for the 400 mg once daily, q12h, and q8h cohorts, respectively. Cis-ceftibuten and trans-ceftibuten recovery in urine was 47% and 6%, respectively, following a single dose of 1,200 mg.

**CLINICAL TRIALS:**

This study is registered with ClinicalTrials.gov as NCT04314206.

## INTRODUCTION

Ceftibuten (Cedax), a broad-spectrum, third-generation, highly bioavailable oral cephalosporin antibiotic active against multiple gram-positive and -negative pathogens, was approved by the United States Food and Drug Administration in 1995 for the treatment of acute bacterial exacerbations of chronic bronchitis in adults and children aged 6 months or older (1) but is no longer available in the United States due to lack of a commercial market. Ceftibuten is approved and marketed in several European countries for the treatment of upper respiratory tract infections, lower respiratory tract infections, and urinary tract infections in adults and children over 6 months of age (2).

The increasing global incidence of antibiotic resistance in Enterobacterales has created an unmet medical need for new oral agents to treat serious infections caused by extended-spectrum β-lactamases (ESBLs) producing Enterobacterales and carbapenem-resistant Enterobacterales (3, 4). Due to its broad spectrum of activity, ceftibuten is an appealing candidate for development in combination with a β-lactamase inhibitor that can restore activity against drug-resistant Enterobacterales. Ledaborbactam etzadroxil (previously VNRX-7145) is the prodrug of the active broad-spectrum β-lactamase inhibitor ledaborbactam, which is capable of inhibiting serine β-lactamases, including Ambler class A ESBLs and KPC-type carbapenemases, class C cephalosporinases, and class D oxacillinases, including the OXA-48 carbapenemase. Ceftibuten–ledaborbactam etzadroxil is being developed to address the unmet medical need for oral antibiotics to treat serious infections, including complicated urinary tract infections (cUTI). Here we report a phase 1 study conducted to evaluate the safety and pharmacokinetics (PK) of ceftibuten alone at doses higher than the approved 400 mg once-daily regimen, which will be required in combination with ledaborbactam etzadroxil for the treatment of Enterobacterales infections.

## RESULTS

### Disposition and participant demographics

A total of 36 participants (*n* = 12 per cohort [ceftibuten, *n* = 9; placebo, *n* = 3]) were enrolled in the study. Thirty-five participants completed the study per the protocol. One participant randomized to receive ceftibuten 1,200 mg on Day 1 and 400 mg every 8 hours (q8h) on Days 3–12 (1,200 mg cohort) discontinued study treatment and the study on Day 3 due to an adverse event (positive COVID-19 test result) and was not replaced.

Participant demographics and baseline characteristics were similar across cohorts (Table 1). Distributions of participant sex and weight varied across dose groups, with a lower mean weight of the subjects in the ceftibuten 1,200 mg cohort likely due to the distribution of females to males in that cohort (89% females in the ceftibuten 1,200 mg cohort versus relatively equal distribution in the other cohorts).

**TABLE 1:** Participant demographics and baseline characteristics^*a*^

	Ceftibuten 400 mg cohort (*n* = 9)	Ceftibuten 800 mg cohort (*n* = 9)	Ceftibuten 1,200 mg cohort (*n* = 9)	Pooled placebo cohorts (*n* = 9)	Overall (*n* = 36)
Age, years, mean (SD)	29.8 (5.3)	28.2 (7.8)	28.4 (9.7)	24.3 (3.8)	27.7 (7.0)
Male	5 (56)	4 (44)	1 (11)	4 (44)	14 (39)
Race					
White	6 (67)	6 (67)	6 (67)	9 (100)	27 (75)
Black or African American	2 (22)	1 (11)	0	0	3 (8)
Asian	0	0	2 (22)	0	2 (6)
Other^*b*^	1 (11)	2 (22)	1 (11)	0	4 (11)
Not Hispanic or Latino	9 (100)	8 (89)	9 (100)	9 (100)	35 (97)
Height, cm, mean (SD)	177.9 (8.7)	172.1 (12.9)	168.0 (8.6)	176.6 (6.5)	173.6 (9.9)
Weight, kg, mean (SD)	73.5 (8.1)	70.80 (13.3)	67.3 (7.6)	73.2 (14.9)	71.2 (11.2)
BMI, kg/m^2^, mean (SD)	23.3 (2.6)	23.7 (1.6)	23.8 (1.9)	23.3 (3.4)	23.5 (2.4)

^*a*^ Except where otherwise specified, data are *n* (%). SD, standard deviation.

^*b*^ Participants categorized as “other” self-identified as “White and Black or African American” or as “White and Asian”.

### Safety and tolerability

Treatment-emergent adverse events (TEAEs) were reported for 69% of participants (ceftibuten, 67%; placebo, 78%) (Table 2), across the combined single- and multiple-dose periods. In the single-dose period, 47% of participants (ceftibuten, 48%; placebo, 44%) experienced TEAEs. In the multiple-dose period, 50% of participants (ceftibuten, 48%; placebo, 56%) experienced TEAEs. No clinically relevant trends in TEAEs were identified between ceftibuten- and placebo-treated participants, nor among participants in the three ceftibuten dose cohorts. Gastrointestinal TEAEs were similar in the combined analysis, both between the pooled ceftibuten group (33%) and the placebo group (33%).

**TABLE 2:** Summary of treatment-emergent adverse events (TEAEs) occurring in >10% of ceftibuten-exposed participants in the combined study periods^*a*^

	Pooled placebo cohorts (*n* = 9)	Pooled ceftibuten cohorts (*n* = 27)	Total (*n* = 36)
Participants with any TEAE	7 (78)	18 (67)	25 (69)
General disorders and administration site conditions	6 (67)	9 (33)	15 (42)
Fatigue	1 (11)	4 (15)	5 (14)
Gastrointestinal disorders	3 (33)	9 (33)	12 (33)
Nausea	1 (11)	6 (22)	7 (19)
Diarrhea	2 (22)	3 (11)	5 (14)
Abdominal pain	0	3 (11)	3 (8)
Nervous system disorders	3 (33)	4 (15)	7 (19)
Headache	3 (33)	4 (15)	7 (19)

^*a*^ Data are number (%) of participants.

The most common TEAEs among participants randomized to ceftibuten were nausea (22%), headache (15%), and fatigue (15%). All TEAEs were mild in severity, except for a moderate TEAE of headache in one placebo group participant. No clinically relevant changes in post-baseline vital signs or clinical laboratory, ECG, or physical examination findings were identified. No serious adverse events, drug-related study discontinuations, or deaths occurred.

### Pharmacokinetics

#### Single-dose pharmacokinetics

##### Plasma results

**Fig 1:**
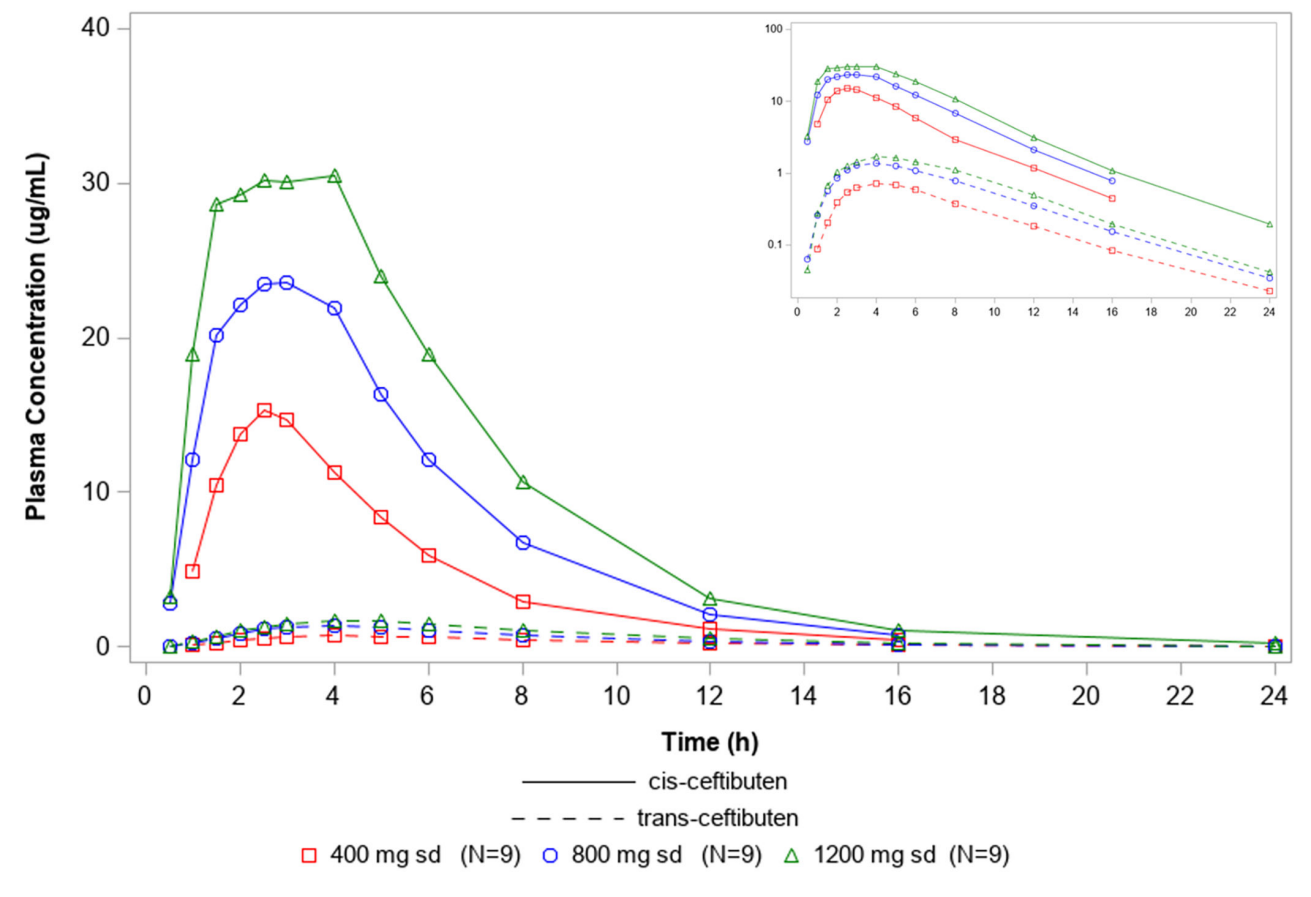
Mean cis-ceftibuten (solid lines) and trans-ceftibuten (dashed lines) plasma concentrations versus time (linear scale) following single doses of ceftibuten. The inset shows a logarithmic concentration scale.

Arithmetic mean plasma concentration–time profiles for cis-ceftibuten and trans-ceftibuten following administration of single doses of ceftibuten 400–1,200 mg are shown in Fig. 1. After administration of a single ceftibuten dose of 400–1,200 mg, for cis-ceftibuten, the geometric mean half-life (*t*_1/2_) was 2.7–2.9 hours and geometric mean clearance (CL/F) was 5.30–5.91 L/h; for trans-ceftibuten, the geometric mean apparent *t*_1/2_ was 3.3–3.5 hours (Table 3). Cis-ceftibuten AUC_inf_ increased dose proportionally, from 75.5 h·µg/mL in the ceftibuten 400 mg cohort to 203 h·µg/mL in the ceftibuten 1,200 mg cohort (slope, 0.90 [95% CI, 0.75–1.05]). *C*_max_ increased less than proportionally with increasing ceftibuten single doses (slope for cis-ceftibuten, 0.68 [95% CI, 0.57–0.80]).

**TABLE 3:** Geometric mean (% geometric coefficient of variation) of cis-ceftibuten and trans-ceftibuten pharmacokinetic parameters following single-dose (day 1) and multiple-dose (day 12) oral administration of ceftibuten

Analyte	Pharmacokinetic parameter^*a*^	Ceftibuten 400 mg cohort	Ceftibuten 800 mg cohort	Ceftibuten 1,200 mg cohort
Day 1^*b*^		400 mg (*n* = 9)	800 mg (*n* = 9)	1,200 mg (*n* = 9)
Cis-ceftibuten	*C*_max_ (µg/mL)	16.9 (14.5)	27.5 (18.4)	35.8 (16.3)
	*T*_max_ ^*c*^	2.5 (1.5–4.0)	2.1 (1.5–4.0)	2.5 (1.5–4.0)
	*t*_1/2_ (h)	2.9 (22.0)	2.8 (23.5)	2.7 (22.7)
	AUC_inf_ (h·µg/mL)	75.5 (21.0)	143 (21.1)	203 (22.0)
	CL/F (L/h)	5.30 (21.0)	5.61 (21.1)	5.91 (22.0)
	Vz/F (L)	22.1 (22.1)	22.5 (24.3)	22.6 (24.0)
Trans-ceftibuten	*C*_max_ (µg/mL)	0.753 (18.7)	1.45 (26.1)	1.75 (18.9)
	*T*_max_ ^*c*^	4.0 (2.0–5.0)	4.0 (2.5–5.0)	4.0 (3.0–5.0)
	*t*_1/2_ (h)	3.52 (21.1)	3.46 (15.6)	3.30 (11.5)
	AUC_inf_ (h·µg/mL)	5.64 (22.4)	11.0 (26.5)	14.2 (26.9)

Day 12^*d*^		400 mg once daily (*n* = 9)	400 mg q12h (*n* = 9)^*e*^	400 mg q8h (*n* = 8)^*e*^
Cis-ceftibuten	C_max_ (µg/mL)	18.2 (19.1)	19.1 (15.6)	24.7 (15.5)
	*T*_max_ ^*d*^	2.0 (1.5–4.0)	3.0 (2.0–5.0)	3.0 (2.0–4.0)
	*t*_1/2_ (h)	2.68 (14.2)	2.52 (18.0)	2.80 (26.7)
	AUC_tau_ (h·µg/mL)	82.4 (19.3)	85.9 (21.0)	105 (16.3)
	R_ac_^*f*^	1.06 (7.0)	1.09 (18.8)	1.24 (14.4)
	CLss/F	4.86 (19.3)	4.66 (21.0)	3.81 (16.3)
	Vz/F	18.8 (14.7)	16.9 (16.3)	15.4 (20.2)
Trans-ceftibuten	*C*_max_ (µg/mL)	0.756 (21.1)	0.970 (20.7)	1.28 (27.4)
	*T*_max_ ^*c*^	4.0 (2.7–6.0)	4.0 (3.0–6.0)	4.0 (3.0–5.0)
	*t*_1/2_ (h)	3.57 (13.1)	3.44 (13.3)	3.61 (12.9)
	AUC_tau_ (h·µg/mL)	5.80 (23.2)	6.25 (23.4)	7.27 (28.8)
	R_ac_^*f*^	1.01 (8.3)	1.22 (24.4)	1.56 (19.0)

^*a*^ AUC_inf_, area under the plasma concentration–time curve from time 0 through infinity; AUC_tau_, area under the concentration–time curve from time of administered dose through dosing interval (daily, tau = 24 hours; q12h, tau = 12 hours; q8h: tau = 8 hours); CL/F, apparent total plasma clearance (cis-isomer only); CLss/F, apparent total plasma clearance at steady state (cis-isomer only); *C*_max_, maximum plasma concentration; *t*_1/2_, terminal half-life; Vz/F, apparent volume of distribution at terminal phase (cis-isomer only).

^*b*^ Parameters assessed following a single dose.

^*c*^ *T*_max_ values are median hours (range).

^*d*^ Parameters assessed on day 12, after 10 consecutive days of dosing.

^*e*^ q12h, every 12 hours; q8h, every 8 hours.

^*f*^ R_ac_ calculated as AUC_tau_ day 12/day 3.

##### Urine results

Urinary pharmacokinetics were assessed only in the ceftibuten 1,200 mg cohort. During the 24-hour period after a single dose on day 1, approximately 53% of the administered dose was recovered in urine: for cis-ceftibuten, Fe was 47.0%, and the mean renal clearance (CL_R_) was 2.76 L/h; for trans-ceftibuten, Fe was 6%, and mean CL_R_ was 5.07 L/h.

### Multiple-dose pharmacokinetics

#### Plasma results

**Fig 2:**
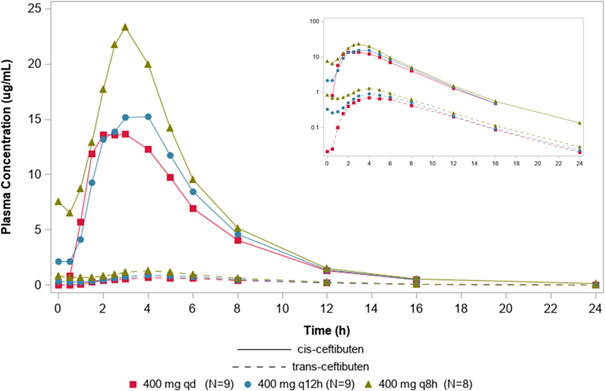
Mean cis-ceftibuten (solid lines) and trans-ceftibuten (dashed lines) plasma concentrations versus time (linear scale) following 10 days of dosing of ceftibuten. The inset shows a logarithmic concentration scale.

Arithmetic mean plasma concentration–time profiles for cis-ceftibuten and trans-ceftibuten following administration of multiple doses of ceftibuten mg are shown in Fig. 2. In the multiple-dose period, steady-state plasma concentrations of cis-ceftibuten were reached within 1 day in all cohorts. The arithmetic mean trough concentrations increased with increasing dose. Across cohorts, on day 12, the cis-ceftibuten geometric mean *t*_1/2_ was 2.5–2.8 hours, and the geometric mean accumulation ratio (R_ac_) was 1.06–1.24 (Table 3). The geometric mean steady-state clearance (CL_SS_/F) decreased slightly with increasing total daily dose, from 4.86 L/h in the 400 mg once daily cohort to 3.81 L/h in the 400 mg q8h cohort. For trans-ceftibuten, across cohorts, the geometric mean *t*_1/2_ was 3.4–3.6 hours, and R_ac_ was 1.01–1.56 (Table 3).

### Protein binding

The geometric mean percentage of free drug (unbound to protein) was approximately 40% (range, 34.8%–44.5%) for both cis-ceftibuten and trans-ceftibuten and did not appear to be dose dependent.

## DISCUSSION

Ledaborbactam etzadroxil is an investigational broad-spectrum boronate β-lactamase inhibitor with the potential to restore the activity of ceftibuten against Enterobacterales, including those producing serine β-lactamases, and to address the need for oral antibiotics to treat drug-resistant Enterobacterales infections. The European Committee on Antimicrobial Susceptibility Testing (EUCAST) has set the ceftibuten susceptibility breakpoint for Enterobacterales at a minimum inhibitory concentration of ≤1 µg/mL for infections originating in the urinary tract (5). In a collection of 1,066 urinary isolates, ceftibuten alone and ceftibuten-ledaborbactam inhibited 25.5% and 89.1%, respectively, of Enterobacterales with a MIC of ≤1 µg/mL (6). Ceftibuten-ledaborbactam also inhibited 89.7%, 98.3%, and 54.1% of multidrug-resistant, phenotypic ESBL, and carbapenem-nonsusceptible Enterobacterales, respectively (7).

The safety findings of this study indicated that single doses of ceftibuten up to 1,200 mg and multiple doses up to 400 mg q8h for 10 days were safe and well tolerated, with a consistent safety profile at doses exceeding the approved 400 mg daily dose. The known safety profile for ceftibuten includes the following common (>1%) adverse reactions: nausea (4%), headache (3%), diarrhea (3%), dyspepsia (2%), dizziness (1%), abdominal pain (1%), and vomiting (1%) (1). With the exception of dizziness, all these commonly labeled adverse reactions were reported for participants in the ceftibuten cohorts of the current study. An important observation was that although gastrointestinal adverse events predominate among the labeled adverse reactions and the adverse events reported in this study, the incidence of gastrointestinal TEAEs did not increase at ceftibuten doses higher than 400 mg daily when dosed for 10 days.

The vast majority of ceftibuten that is dosed is cis-ceftibuten. Following dosing, ceftibuten undergoes isomerization to trans-ceftibuten; approximately 10% of ceftibuten is converted to the trans-ceftibuten isomer, which has about one-eighth the antimicrobial potency of the cis-ceftibuten isomer (1). Consistent with the known pharmacokinetic characteristics of ceftibuten, in our study, cis-ceftibuten was the predominant isomer in plasma; exposures of the trans-ceftibuten isomer represented <10% of the total exposure. The current findings generally align with the prior studies of single and multiple doses of ceftibuten 400 mg. After a single dose, mean cis-ceftibuten area under the curve (AUC) has been reported as 73.7–95.3 µg•h/mL, *C*_max_ as 15.0–17.6 µg/mL, *T*_max_ as 2.0–2.6 hours, and *t*_1/2_ as 2.3–2.5 hours (8–12). After multiple doses (8–10 days) of ceftibuten 400 mg bid, mean cis-ceftibuten AUC has been reported as 97.1–118.6 μg•h/mL, *C*_max_ as 20.6–21.7 µg/mL, *T*_max_ as 2.3–2.4 hours, and *t*_1/2_ as 2.4–3.1 hours (8, 10). Plasma protein binding was also similar to previously reported values (1). Dose proportionality was observed for AUC but was less than dose proportional for *C*_max_, following single doses of 400–1,200 mg. Loss of dose proportionality has been observed for ceftibuten *C*_max_ and AUC when doses greater than 400 mg are administered, suggesting that absorption may be saturated in the proximal gastrointestinal tract (8, 10, 12).

The absolute bioavailability of ceftibuten is unknown; however, measurements of cis-ceftibuten in urine following 400 mg dose(s) suggest at least 50% bioavailability (9, 11, 12). Total urinary recovery within 24 hours of the administered cis-ceftibuten dose in this study was approximately 47% in the 1,200 mg cohort. Urinary excretion was not measured in lower dose cohorts and prevents direct comparison to previous studies. In one recent study, urinary recovery of cis-ceftibuten was 83% and 87% following single doses of ceftibuten 400 and 600 mg, respectively, but was 64% after a single dose of ceftibuten 800 mg (10). The lower urinary recovery of 47% following a single dose of ceftibuten 1,200 mg in the current study may be due in part to a shorter collection period relative to the study by Hernández-Mitre et al. (collection over 24 hours versus 48 hours) (10). In a study by Bressolle et al., in which participants received ceftibuten 400 mg q12h for 7 days, urinary recovery of cis-ceftibuten was 58% following a single dose of ceftibuten and a 12-hour collection period and increased to 63% following the day 8 dose and a 48-hour collection period (9). In addition, lower recovery at a 1,200 mg dose might reflect an elimination pathway that is subject to saturation at higher doses (10, 12).

In conclusion, this study in healthy adults demonstrated that higher doses of ceftibuten were safe and well tolerated. Ceftibuten is a highly bioavailable oral cephalosporin, and the pharmacokinetic profile at higher doses is favorable and supports the higher doses required to treat drug-resistant Enterobacterales infections. This study supports the continued evaluation of ceftibuten–ledaborbactam etzadroxil as an oral treatment for serious infections, includingcUTI.

## MATERIALS AND METHODS

### Study design

This phase 1, two-period, randomized, double-blind, placebo-controlled, sequential-group, ascending dose study of ceftibuten administered orally for 10 days in healthy adults was conducted at a single site (PRA Health Sciences; Groningen, The Netherlands). The study protocol was reviewed and approved by an institutional review board, and the study was done in accordance with the ethical principles that have their origin in the Declaration of Helsinki, with the International Council for Harmonisation Guideline for Good Clinical Practice, and with all applicable regulatory requirements. All participants provided written informed consent before being screened. The study is registered on ClinicalTrials.gov (NCT04314206).

Eligible participants were randomized at a 3:1 ratio to receive ceftibuten or placebo. Participants were enrolled in one of three cohorts; each cohort comprised 12 participants, nine randomized to ceftibuten and three randomized to placebo. Within each cohort, a single oral dose was administered on day 1 (single-dose period), followed by a 1-day washout period and 10 days of repeated oral doses (days 3–12; multiple-dose period). Participants in the 400 mg cohort received a single dose of ceftibuten 400 mg or placebo during the single-dose period and ceftibuten 400 mg or placebo once daily during the multiple-dose period. Participants in the 800 mg cohort received a single dose of ceftibuten 800 mg or placebo during the single-dose period and ceftibuten 400 mg every 12 hours (q12h; total daily dose 800 mg) or placebo during the multiple-dose period. Participants in the 1,200 mg cohort received ceftibuten 1,200 mg or placebo during the single-dose period and ceftibuten 400 mg q8h (total daily dose 1,200 mg) or placebo during the multiple-dose period. On day 12, participants in the 400 mg q12h and 400 mg q8h cohorts received only a single morning dose.

After completion of the 400 and 800 mg cohorts, a safety monitoring committee reviewed all available blinded safety data before the decision was made to begin dosing in the 1,200 mg cohort.

### Participants

Healthy men and nonpregnant women, 18–55 years of age, with a body mass index of ≥18.5 kg/m^2^ and ≤30.0 kg/m^2^ and normal vital signs, hematology, and clinical chemistry values were eligible to enroll. Key exclusion criteria were regular use of medication for a chronic condition, history of drug allergy requiring urgent medical treatment, hypersensitivity to a β-lactam antibacterial drug, positive drug screen, confirmed or suspected COVID-19 infection, or positive screening tests for hepatitis B surface antigen, anti-hepatitis C virus antibodies, or anti-human immunodeficiency virus 1 or 2 antibodies. Participants were required to discontinue any medications, vitamins, supplements, nicotine, and vaping products at least 14 days prior to dosing; exceptions were acetaminophen at doses ≤ 3 g/day and hormonal contraceptives.

### Assessments

Safety was assessed via ongoing monitoring of adverse events and changes in physical examination findings, clinical laboratory measures, vital signs, and ECGs. For determination of single-dose and steady-state plasma pharmacokinetics of cis- and trans-ceftibuten, serial blood samples were collected pre-dose and 0.5, 1, 1.5, 2, 2.5, 3, 4, 5, 6, 8, 12, 16, and 24 hours after the day 1 dose; pre-dose and 0.5, 1, 1.5, 2, 2.5, 3, 4, 5, 6, 8, 12 (400 mg daily and 400 mg q12h cohorts), and 16 hours (400 mg daily cohort) after the day 3 dose; pre-dose on days 4, 6, 8, and 10; and pre-dose and 0.5, 1, 1.5, 2, 2.5, 3, 4, 5, 6, 8, 12, 16, and 24 hours after the day 12 dose. Additional blood samples were collected at 2, 4, and 8 hours post-dose on days 1 and 12 for the evaluation of cis-ceftibuten and trans-ceftibuten protein binding. For assessment of cis-ceftibuten and trans-ceftibuten urine pharmacokinetics, urine samples were collected within 12 hours before the day 1 dose; all urine was collected predose and for 24 hours following dosing on days 1 and 12.

### Sample collection and analysis

For plasma pharmacokinetics and protein binding assays, 6 (pharmacokinetics analyses) or 10 mL (protein binding) blood was collected in prelabeled, prechilled 6 or 10 mL K_2_EDTA collection tubes (BD Biosciences). Tubes were inverted 8–10 times, placed on ice, centrifuged for 10 minutes at 1,000 × *g* at 4°C, and stored ≤60 minutes after sample collection at –70°C or lower. For urine pharmacokinetics assays, urine samples comprised 2 mL of the total urine volume collected from participants during each collection period. Urine samples were transferred into a prechilled 15 mL conical storage tube (ThermoFisher Scientific) and stored upright, ≤120 minutes after the end of each collection period, at –70°C or lower.

Plasma and urine samples were assayed for cis-ceftibuten and trans-ceftibuten using bioanalytical methods that met all validation criteria for accuracy, precision, and bias. Liquid chromatography–tandem mass spectrometry (LC–MS/MS) was used to analyze plasma samples for cis-ceftibuten using a calibration standard of 0.150–75.0 ug/mL based on the analysis of 0.100 mL of mixed matrix plasma, and for trans-ceftibuten using a calibration standard of 8.00–4,000 ng/mL based on the analysis of 0.100 mL of mixed matrix plasma. Mixed matrix plasma containing cis-ceftibuten and trans-ceftibuten and the internal standard ^13^C_3_,^15^N_2_-ceftibuten (a mixture of cis-ceftibuten and trans-isomers) was extracted using protein-precipitate plus lipid filter and analyzed using Sciex API 5000 LC-MS/MS with an HPLC column. The peak area of the *m*/*z* 411.3 → 268.1 cis-ceftibuten and trans-ceftibuten product ion was measured against the peak area of the *m*/*z* 416.0 → 273.0 ^13^C_3_,^15^N_2_-cis-ceftibuten and ^13^C_3_,^15^N_2_-trans-ceftibuten internal standard product ion. Quantitation used a weighted 1/x^2^ linear least-squares regression analysis generated from calibration standards that were prepared on the day of extraction.

Plasma protein binding for cis- and trans-ceftibuten was determined using ultracentrifugation and assays for total and unbound concentrations.

LC–MS/MS was used to analyze urine samples for cis-ceftibuten using a calibration standard of 0.0600–30.0 ug/mL based on the analysis of 0.200 mL of urine, and for trans-ceftibuten using a calibration standard of 3.00–1,500 ng/mL based on the analysis of 0.200 mL of urine.

The characteristics of the bioanalytical assays for cis- and trans-ceftibuten in plasma and urine are provided in Table S2.

### Pharmacokinetic and statistical analyses

The plasma PK samples collected on days 1, 3, and 12 were used to generate full pharmacokinetic profiles; the remaining plasma pharmacokinetic samples were used to determine trough plasma pharmacokinetic samples prior to dosing on days 4, 6, 8, 10, and 12. Plasma and urine pharmacokinetic parameters were calculated using noncompartmental analysis of the plasma and urine concentration–time data for cis-ceftibuten and trans-ceftibuten (Certara WinNonlin, Radnor, PA). Urine samples were analyzed for the 1,200 mg cohort only.

Single-dose plasma pharmacokinetic parameters were determined in the single-dose period, and both single-dose (day 1) and multiple-dose (day 12) pharmacokinetic parameters were determined in the multiple-dose period. Estimated pharmacokinetic parameters included *C*_max_, time to *C*_max_ (*T*_max_), AUC_tau_, AUC_inf_, *t*_1/2_, CL/F, *V*z, F_e_, and CL_R_. For multiple-dose regimens, the accumulation ratio (R_ac_) was calculated as AUC_tau_ day 12 (steady state)/AUC_tau_ day 3 (first dose). The actual sample times were used to calculate the pharmacokinetic parameters. Calculation of *λ*_Z_ was based on the best fit of concentrations in the observed terminal elimination phase of a profile, and statistical inclusion of any parameter based on *λ*_Z_ required the goodness-of-fit statistic (*R*^2^) to be greater than 0.8. Dose proportionality based on *C*_max_ and AUC were compared across dose levels on days 1 and 12. Dose proportionality was determined via linear regression analysis using the following formula: ln(PK) = ln(β0) + β1 ln(Dose) + ε, for which PK was the pharmacokinetic parameter tested (e.g., *C*_max_ or AUC), ln(β0) was the *y* intercept, β1 was the slope, and ε was an error term. A slope with a 90% CI containing 1 indicated linearity or dose proportionality. Dose proportionality was assessed for the single-dose period only, as all cohorts received a single 400 mg morning dose of ceftibuten on day 12 of the multiple-dose period.

As per protocol, urine samples were analyzed for only the 1,200 mg cohort, a higher dose than those for which urine PK data have not previously been reported. Urinary analyses determined the amount of cis-ceftibuten and trans-ceftibuten excreted in urine over time, the percentage eliminated renally, and an estimate of renal clearance (CL_R_).

Pharmacokinetic parameters were analyzed for all participants who received at least one dose of study drug and had evaluable pharmacokinetic parameters for the single-dose or multiple-dose periods. Adverse events and other safety parameters were summarized using descriptive statistics for all participants who received at least one dose of study drug in the single-dose or multiple-dose periods. Continuous variables were summarized using descriptive statistics by treatment, including *n*, arithmetic mean, standard deviation, median, and range. For pharmacokinetic data, the coefficient of variation (%CV), geometric mean, and geometric CV were also calculated.

## SUPPLEMENTAL MATERIAL

Supplemental tables aac.00087-25-s0001.docx Tables S1 and S2.

ASM does not own the copyrights to Supplemental Material that may be linked to, or accessed through, an article. The authors have granted ASM a non-exclusive, world-wide license to publish the Supplemental Material files. Please contact the corresponding author directly for reuse.
