# Supplementary material for: Pharmacokinetics and safety of single and repeat doses of ceftibuten in healthy participants: a phase 1 dose escalation study
Source: Antimicrob Agents Chemother. 2025 Aug 5;69(9):e00087-25. doi: 10.1128/aac.00087-25 (PMC12406654; doi:10.1128/aac.00087-25)
Supplement: Supplemental tables — Tables S1 and S2. [file aac.00087-25-s0001.docx]

# Supplemental Tables

Table S1. Summary of treatment-emergent adverse events (TEAEs) occurring in >10% of ceftibuten-exposed participants overall, by study period and dose.

| **Period 1: Single Dose** | **Placebo (n = 9)** | **Ceftibuten^a^** | | | | **Overall (N = 36)** |
| --- | --- | --- | --- | --- | --- | --- |
|  |  | **400 mg  (n = 9)** | **800 mg (n = 9)** | **1200 mg  (n = 9)** | **Total (n = 27)** |  |
| Any TEAE | 4 (44) | 3 (33) | 3 (33) | 7 (78) | 13 (48) | 17 (47) |
| Gastrointestinal disorders | 1 (11) | 1 (11) | 1 (11) | 3 (33) | 5 (19) | 6 (17) |
| Nausea | 0 | 1 (11) | 1 (11) | 1 (11) | 3 (11) | 3 (8) |
| Diarrhea | 0 | 0 | 0 | 1 (11) | 1 (4) | 1 (3) |
| General disorders and administration site conditions | 3 (33) | 2 (22) | 2 (22) | 1 (11) | 5 (19) | 8 (22) |
| Fatigue | 0 | 0 | 1 (11) | 0 | 1 (4) | 1 (3) |
| Nervous system disorders | 0 | 0 | 1 (11) | 0 | 1 (4) | 1 (3) |
| Headache | 0 | 0 | 1 (11) | 0 | 1 (4) | 1 (3) |
| **Period 2: Multiple Dose** | **Placebo (n = 9)** | **Ceftibuten^a^** | | | | **Overall (N = 36)** |
|  |  | **400 mg  once daily (n = 9)** | **400 mg q12h^a^ (n = 9)** | **400 mg q8h^a^ (n = 9)** | **Total (n = 27)** |  |
| Any TEAE | 5 (56) | 3 (33) | 6 (67) | 4 (44) | 13 (48) | 18 (50) |
| General disorders and administration site conditions | 4 (44) | 1 (11) | 2 (22) | 4 (44) | 7 (26) | 11 (31) |
| Fatigue | 1 (11) | 0 | 2 (22) | 2 (22) | 4 (15) | 5 (14) |
| Nervous system disorders | 3 (33) | 1 (11) | 2 (22) | 0 | 3 (11) | 6 (17) |
| Headache | 3 (33) | 1 (11) | 2 (22) | 0 | 3 (11) | 6 (17) |
| Gastrointestinal disorders | 2 (22) | 2 (22) | 2 (22) | 2 (22) | 6 (22) | 8 (22) |
| Diarrhea | 2 (22) | 1 (11) | 1 (11) | 0 | 2 (7) | 4 (11) |
| Nausea | 1 (11) | 1 (11) | 0 | 2 (22) | 3 (11) | 4 (11) |
| Abdominal pain | 0 | 1 (11) | 0 | 2 (22) | 3 (11) | 3 (8) |

Data are number (%) of participants.

^a^q12h, every 12 hours; q8h, every 8 hours.

Table S2. Summary of bioanalytical assay characteristics.

| Analyte | Cis-ceftibuten | | Trans-ceftibuten | |
| --- | --- | --- | --- | --- |
| Matrix | Plasma | Urine | Plasma | Urine |
| Platform^a,b^ | LC-MS/MS equipped with HPLC column | LC-MS/MS equipped with HPLC column | LC-MS/MS equipped with HPLC column | LC-MS/MS equipped with HPLC column |
| Calibration Range | 0.15 to 75 µg/mL | 0.06 to 30 ug/mL | 0.008 to 4 µg/mL | 0.003 to 1.5 µg/mL |
| Standard Calibration Curve Performance |  |  |  |  |
| Cumulative accuracy (%bias) | -4.3% to 2.9% | -6.3% to 4.7% | -8.5% to 6.5% | -4.7% to 4.3% |
| Cumulative Precision (%CV) | ≤ 3.8% | ≤ 2.6% | ≤ 2.9% | ≤ 3.4% |
|  |  |  |  |  |
| QC Performance |  |  |  |  |
| Cumulative Accuracy (%bias) | -1.2% to 6.6% | -5.4% to 2.0% | -2.2% to 10.0% | -5.0% to 6.0% |
| Interbatch %CV | ≤ 8.7% | ≤ 3.5% | ≤ 3.6% | ≤ 9.7% |

^a^LC-MS/MS, liquid chromatography – tandem mass spectrometry; HPLC, high performance liquid chromatography.

^b^LC-MS/MS (Sciex API 5000) equipped with HPLC column (Ace 3 C18-PFP, 150 x 2.1 mm)
